# Effects of different doses of esketamine as an adjunct to sufentanil-based patient-controlled intravenous analgesia on postoperative analgesia and opioid consumption following lumbar decompression surgery: a dose-finding randomized controlled trial

**DOI:** 10.3389/fphar.2026.1946726

**Published:** 2026-10-14

**Authors:** Jun-jie Liu, Xin Wang, Pan-pan Ding, Hong-jian Wang, Ting-ting Zhang, Xian-wen Hu

**Affiliations:** 1 Department of Anesthesiology, The Second Affiliated Hospital of Anhui Medical University, Hefei, Anhui, China; 2 Department of Anesthesiology, The Second People’s Hospital of Hefei, Hefei Hospital Affiliated to Anhui Medical University, Hefei, Anhui, China

**Keywords:** adverse drug reactions, dose-finding, esketamine, lumbar decompression surgery, opioid-sparing, oxidative stress, postoperative analgesia, randomized controlled trial

## Abstract

**Objective:**

This study aimed to evaluate the effects of different doses of esketamine as an adjunct to sufentanil-based patient-controlled intravenous analgesia (PCIA) on postoperative analgesia and opioid consumption after lumbar decompression surgery and to explore its optimal risk–benefit dose.

**Methods:**

A total of 120 patients scheduled for lumbar decompression surgery were randomly allocated to four groups. The control group (Group N, n = 30) received PCIA with sufentanil 2.5 μg/kg. To test the opioid-sparing hypothesis, the three experimental groups (Groups S1, S2, and S3, n = 30 each) received PCIA with a lower sufentanil dose (1.5 μg/kg) combined with esketamine at 0.25, 0.5, and 1.0 mg/kg, respectively. All PCIA solutions were diluted with normal saline to a total volume of 100 mL. The primary outcome was the Visual Analogue Scale (VAS) pain score at rest. Secondary outcomes included sufentanil consumption, PCIA press counts, requirement for rescue analgesia, Ramsay Sedation Scale (RSS) scores, and oxidative-stress markers; safety endpoints were analyzed as exploratory outcomes. Postoperative adverse effects, including nausea, vomiting, dizziness, respiratory depression, hypotension, and psychiatric symptoms (dissociation or nightmares), were recorded and compared across the four groups.

**Results:**

All esketamine-containing regimens (S1–S3) showed significantly lower VAS scores at all postoperative time points compared with Group N (P < 0.05), with no statistically significant difference among S1, S2, and S3. The 24-h sufentanil consumption, effective PCIA presses, and rescue analgesia demand were also significantly reduced (P < 0.05). However, Ramsay sedation scores increased dose-dependently (S3 > S2 > S1). At 4 h postoperatively, SOD activity was higher, and concentrations of MDA, GLU, and COR were lower in all esketamine-containing regimens compared with Group N. Although the overall incidence of adverse events was comparable, the high-dose group (S3) had a significantly increased incidence of hypotension and dissociative symptoms.

**Conclusion:**

Adding esketamine to a reduced-sufentanil PCIA regimen improved postoperative analgesia and reduced opioid consumption after lumbar decompression surgery. Analgesic benefit was similar across the tested doses (0.25–1.0 mg/kg), whereas dose-related adverse effects (sedation, hypotension, psychomimetic symptoms) increased with dose. These findings suggest that 0.25 mg/kg may provide a favorable efficacy-safety balance within the investigated dose range.

**Clinical Trial registration:**

http://www.chictr.org.cn/showproj.aspx?proj=249890, Identifier: ChiCTR2500095890.

## 1 Introduction

Lumbar decompression surgery is a common spinal procedure primarily used to alleviate symptoms of nerve root compression caused by lumbar disc herniation or spinal stenosis. This surgical intervention is often accompanied by considerable intraoperative and postoperative pain. Thus, the implementation of effective analgesic techniques is vital to enhancing patient recovery and improving quality of life (Krauss et al., 2023; Han et al., 2021; Bovonratwet et al., 2022).

Currently, patient-controlled intravenous analgesia (PCIA) is the cornerstone of acute postoperative pain management. However, conventional opioid-based regimens are frequently associated with adverse effects, including postoperative nausea and vomiting (PONV), respiratory depression, gastrointestinal dysfunction (e.g., opioid-induced constipation), and the potential risks of tolerance and addiction (Rajan et al., 2022; Luo et al., 2024).

In recent years, esketamine—the dextrorotatory isomer of ketamine—has garnered significant attention due to its unique antagonistic action on the N-methyl-D-aspartate (NMDA) receptor. This mechanism not only confers its definitive analgesic efficacy but may also positively impact the prevention of chronic postoperative pain by mitigating central sensitization (Kritzer et al., 2022; Johnston et al., 2024; Bodnar et al., 2024). Current research has primarily focused on its application via a single preoperative bolus to prevent hyperalgesia or intraoperative continuous infusion to augment anesthesia and provide early postoperative analgesia (Patzkowski and Patzkowski, 2022; Ding and Yue, 2024; Huang et al., 2023). However, evidence remains insufficient regarding its incorporation as a standard component into postoperative PCIA regimens for systematic and sustained multimodal analgesia.

Theoretically, the addition of low-dose esketamine to a PCIA pump could produce synergistic or additive effects with opioids. This approach may achieve equivalent or superior analgesia while significantly reducing opioid requirements and their associated adverse reactions—a phenomenon known as the “opioid-sparing” effect (Reed et al., 2022). A 2023 systematic review and meta-analysis focusing on spinal surgery indicated that perioperative administration of ketamine (including esketamine) may reduce 24-h postoperative opioid consumption in adult patients (Tornøe et al., 2023).

However, the effects of esketamine exhibit a clear dose-dependency. Lower doses might provide inadequate analgesia with fewer side effects, whereas higher doses, while potentially offering enhanced analgesia, carry an increased risk of adverse reactions such as dizziness, excessive sedation, hypertension, and psychomimetic symptoms (e.g., dissociation) (Yang et al., 2023). Currently, there is a lack of high-level evidence regarding the optimal dosing regimen of esketamine combined with opioids in PCIA following lumbar decompression surgery, and clinical controversy persists.

To address this evidence gap, we conducted a randomized controlled trial to assess and compare the efficacy and safety of three distinct dose regimens of esketamine (0.25, 0.5, and 1.0 mg/kg) as an adjuvant to a reduced-sufentanil PCIA following lumbar decompression surgery. Through a comprehensive analysis of analgesic outcomes (including VAS scores and supplemental opioid consumption), depth of sedation, perioperative stress markers, and adverse event profiles across groups, this study aims to characterize the dose-related efficacy and safety profile of esketamine and to explore the corresponding risk–benefit balance in this specific clinical setting.

The inclusion criteria encompassed: (1) patients slated for lumbar decompression fusion and internal fixation surgery, with an American Society of Anesthesiologists (ASA) classification of I–II; (2) individuals aged 40–65 years; (3) those with normal communication abilities; and (4) written informed consent obtained from all participants.

The exclusion criteria included: (1) severe respiratory or circulatory conditions, such as coronary atherosclerosis, acute myocardial infarction, respiratory failure, or other comorbid diseases; (2) neurological disorders, including severe sequelae of cerebral hemorrhage or infarction, hemiplegia, or aphasia; (3) mental and psychological illnesses, including schizophrenia and depression; (4) long-term analgesic abuse or chronic pain history; (5) significant liver or renal dysfunction; and (6) pregnancy or breastfeeding.

The elimination criteria were: (1) the occurrence of drug allergies or severe adverse events that, in the physician’s opinion, warranted discontinuation of the trial; (2) the necessity for intraoperative expansion of the surgical scope based on pathological or surgical judgment; (3) poor subject compliance, making it impossible to complete follow-up; and (4) detection of major violations of inclusion or exclusion criteria post-randomization.

## 2 Materials and methods

### 2.1 Study design and Ethics

This study was a randomized, double-blind, active-controlled, four-arm parallel-group trial, which was approved by the Ethics Committee of the Second People’s Hospital of Hefei (PKUSSIRB-202486041, 12 November 2024) and prospectively registered with the Chinese Clinical Trial Registry (ChiCTR2500095890, 15 January 2025). The study was conducted at the Second People’s Hospital of Hefei (Anhui, China) in accordance with the CONSORT guidelines and the principles of the Declaration of Helsinki. All participants provided written informed consent.

The Visual Analogue Scale (VAS) pain score at rest was pre-specified as the sole primary outcome; all other efficacy endpoints were secondary outcomes, and safety endpoints were analyzed as exploratory outcomes. We note that the original registry entry temporarily listed several exploratory endpoints under the primary-outcome field owing to a structural constraint of the registration platform at the time of entry. To ensure full compliance with the CONSORT reporting guidelines, the registry has been amended to designate the VAS pain score as the single primary outcome and to reclassify all other endpoints as secondary outcomes. This discrepancy and its correction are disclosed transparently in the Limitations section.

### 2.2 Anesthesia protocol

Upon entering the operating room, the patient’s vital signs were routinely monitored. The patient was positioned supine, and intravenous access was established. Sedation prior to surgery was induced using 1–2 mg of midazolam. Following this, sufentanil at a dosage of 0.2–0.4 μg/kg, propofol at 1.5–2.0 mg/kg, and cisatracurium besylate at 0.1–0.2 mg/kg were administered in sequence. Once adequate muscle relaxation was confirmed, endotracheal intubation was performed, and mechanical ventilation commenced. During surgery, the bispectral index was maintained between 40 and 60 (Zhang et al., 2022). The infusion rates of propofol and remifentanil were titrated based on the patient’s circulatory parameters, and cisatracurium besylate was given intermittently. The routine use of other long-acting analgesic agents was avoided during the intraoperative period. Before skin closure, an additional 5 μg of sufentanil was administered, and propofol infusion was halted. Remifentanil administration was ceased at the conclusion of the surgery. All participants received 10 mg of azasetron intravenously 10 min before the end of the surgery as prophylaxis against postoperative nausea and vomiting. To reverse any remaining neuromuscular blockade, 0.04 mg/kg of neostigmine and 0.02 mg/kg of atropine were administered intravenously. After confirming adequate ventilation, responsiveness to verbal stimuli, and stable tidal volumes, the endotracheal tube was carefully removed, while vital signs and airway patency were closely monitored. After surgery, all patients were transferred to the PACU with PCIA connected, maintaining a VAS score below 3.

To test the opioid-sparing hypothesis, the esketamine-containing regimens used a lower sufentanil dose (1.5 μg/kg) than the control regimen (2.5 μg/kg). Accordingly, differences in analgesic and adverse outcomes reflect the combined effect of esketamine plus reduced sufentanil, and cannot be attributed to esketamine alone.

The PCIA pumps were prepared by an independent anesthesia nurse who was not involved in patient recruitment, intervention administration, or outcome evaluation, according to the randomization codes. Patients, attending anesthesiologists, outcome assessors (trained nurses), and data analysts were all blinded to group allocation. The attending anesthesiologists did not participate in any postoperative assessment; all postoperative outcome measures (VAS, Ramsay Sedation Scale, Riker Sedation–Agitation Scale, and Clinician-Administered Dissociative States Scale) were collected exclusively by trained nurses who were blinded to group assignment.

The PCIA solution was prepared based on body weight–adjusted total doses and diluted to a final volume of 100 mL with 0.9% sodium chloride solution. The control group (Group N) received sufentanil 2.5 μg/kg. The esketamine groups (Groups S1, S2, and S3) received esketamine 0.25, 0.5, and 1.0 mg/kg, respectively, each combined with sufentanil 1.5 μg/kg. The parameters for the PCIA pumps were configured as follows: first dose: 2 mL; background infusion: 2 mL/h; bolus dose: 1 mL; lockout interval: 20 min; maximum limit: 10 mL.

A standardized rescue analgesia protocol was implemented for all patients to manage breakthrough pain. Intravenous flurbiprofen axetil (50 mg) was administered as rescue medication when a patient reported a pain intensity of ≥4 on the Visual Analogue Scale (VAS). Pain scores were assessed at rest every 4 h by nursing staff who were blinded to group allocation. If pain relief was inadequate (VAS remained ≥4) 30 min after the first rescue dose, an additional dose of 50 mg flurbiprofen axetil was given. The total consumption of rescue analgesia within the first 24 postoperative hours was recorded for each patient.

#### 2.2.1 Management of breakthrough pain and psychomimetic adverse events

##### 2.2.1.1 Management of breakthrough (peak) pain

Postoperative pain intensity was assessed at rest every 4 h by blinded nursing staff, and additional assessments were performed whenever a patient reported worsening pain. In anticipation of the peak pain period (typically 4–8 h postoperatively after lumbar decompression surgery), a standardized rescue analgesia protocol was applied: intravenous flurbiprofen axetil 50 mg was administered when the VAS score was ≥4; if pain remained ≥4 30 minutes after the first rescue dose, an additional 50 mg of flurbiprofen axetil was given. The total consumption of rescue analgesia within the first 24 postoperative hours was recorded for each patient.

##### 2.2.1.2 Management of dissociative/psychomimetic symptoms

Because the psychomimetic effects of esketamine are most pronounced during the early postoperative period when plasma concentrations peak, patients were systematically monitored using the Clinician-Administered Dissociative States Scale (CADSS; a score >4 defined a dissociative event). A standardized management strategy was predefined for any patient developing dissociation, nightmares, or agitation: (1) immediate verbal reassurance and placement in a calm, quiet, low-stimulation environment; (2) temporary suspension or dose reduction of the esketamine-containing PCIA infusion; (3) administration of a small dose of a benzodiazepine (midazolam 1–2 mg intravenously) if symptoms persisted, with haloperidol reserved for severe agitation refractory to benzodiazepines; and (4) continuous monitoring of vital signs and level of consciousness until complete resolution of symptoms. Patients who developed severe or persistent psychomimetic symptoms were withdrawn from the esketamine-containing regimen and managed with opioid-based analgesia alone.

### 2.3 Randomized controlled trial

#### 2.3.1 Randomization and blinding

120 patients who underwent planned lumbar decompression surgery were recruited from The Second People’s Hospital of Hefei from 22 January 2025 to 15 October 2025. Patients were randomly allocated to four groups by simple randomization (allocation ratio 1:1:1:1) using a computer-generated random-number sequence, without blocking or stratification. Group N (control): postoperative PCIA solution contained sufentanil 2.5 μg/kg. Group S1: esketamine 0.25 mg/kg plus sufentanil 1.5 μg/kg. Group S2: esketamine 0.5 mg/kg plus sufentanil 1.5 μg/kg. Group S3: esketamine 1.0 mg/kg plus sufentanil 1.5 μg/kg. All PCIA medications were diluted to 100 mL with normal saline. Computer-generated random assignments were placed in sequentially numbered, opaque, sealed envelopes. All envelopes were stored and dispensed by the hospital’s clinical trial pharmacy. On the day of surgery, the envelope was opened by an independent trial coordinator who was not involved in patient recruitment, anesthetic management, or outcome assessment; this individual’s sole role was to reveal the allocation and instruct the independent nurse who prepared the PCIA pump.

This was a double-blind study, in which participants and investigators/outcome assessors were blinded to group allocation. Specifically, patients, attending anesthesiologists, outcome assessors (trained nurses), and data analysts were all blinded; only the independent nurse who prepared the PCIA pumps was unblinded (and was not involved in recruitment, anesthetic management, or outcome assessment). The attending anesthesiologists did not participate in any postoperative outcome assessment; all postoperative assessments were performed exclusively by trained nurses blinded to group allocation.

#### 2.3.2 Observation indicators

The primary outcome of the study was the Visual Analogue Scale (VAS) pain score at rest (Wang D. et al., 2023), assessed at 2, 4, 8, and 12 h postoperatively. The VAS was selected as the primary outcome because it is the most widely used and validated instrument for acute postoperative pain intensity and the standard primary efficacy endpoint in acute postoperative analgesia trials. The Visual Analogue Scale consisted of a 10-cm horizontal line, with the endpoints labeled “No pain/discomfort (0 points)” and “Worst imaginable pain/discomfort (10 points).” Patients marked their perceived level on the line, and the distance (in cm) from the left end to the mark was recorded as the score.

Secondary outcomes encompassed: (1) the total 24-h sufentanil consumption; (2) the number of successful press attempts; (3) the total number of press attempts; (4) the requirement for rescue analgesia; (5) the Ramsay sedation score (Feng et al., 2021) (scores of 2–4 indicate adequate sedation, while scores of 5–6 suggest over-sedation); and (6) oxidative stress markers. For the oxidative stress markers, fasting venous blood samples were drawn from all participants preoperatively and 4 h postoperatively. Following centrifugation at 3,500 rpm for 10 min, the upper serum was stored at a low temperature for further analysis. Superoxide dismutase (SOD) and malondialdehyde (MDA) concentrations were quantified using chemical colorimetry. Blood glucose (GLU) was determined via the oxidase method, and cortisol (COR, nmol/L) levels were measured through radioimmunoassay. The SOD, MDA, GLU, and COR test kits were obtained from Shanghai Jining Biological.

Safety (exploratory) endpoints encompassed: (1) the occurrence of postoperative agitation, nausea, vomiting, drowsiness, and respiratory depression within 24 h after surgery; postoperative agitation was evaluated using the Riker Sedation-Agitation Scale (SAS), with a score of ≥5 defined as agitation; and (2) the frequency of dissociative or nightmare psychiatric symptoms, along with hypotension; dissociative symptoms were evaluated using the Clinician-Administered Dissociative States Scale (CADSS), with a score >4 defined as the presence of a dissociative event. Safety endpoints were analyzed descriptively and are not used to support the primary conclusions.

Sedation was assessed with the Ramsay Sedation Scale, a simple and widely validated instrument for monitoring sedative depth in the perioperative setting; agitation was assessed in parallel using the Riker Sedation-Agitation Scale. We acknowledge that the Richmond Agitation-Sedation Scale (RASS) would provide a more contemporary, unified assessment of sedation and agitation, and this is noted as a limitation.

To ensure unbiased assessment, the Ramsay sedation scores, VAS scores, SAS scores, and CADSS scores were collected by trained nurses who were blinded to the group assignments.

### 2.4 Sample size calculation

The sample size was calculated based on the primary outcome (the VAS pain score at rest) and the primary planned contrast (control vs. esketamine-containing regimens). Based on references (Chen JH. et al., 2024; Chen HZ. et al., 2024), the VAS scores in the control group and the esketamine group were 3.5 ± 1.2 and 2.7 ± 0.8, respectively (an assumed between-group difference of 0.8 points). Using PASS 15.0 software for a one-way ANOVA comparison among four groups, with a two-sided α of 0.05 and a power (1 − β) of 80%, the calculation indicated that approximately 27 patients per group were required. Accounting for an estimated 10% dropout rate, we planned to enroll 30 patients per group, resulting in a total sample size of 120 patients. Because the study employed a single primary outcome, no formal multiplicity adjustment was incorporated into the sample-size calculation; multiplicity was controlled at the analysis stage (Bonferroni correction) for the secondary outcomes.

### 2.5 Statistical analysis

All statistical analyses were performed using SPSS (version 26.0). The normality of the data was assessed using the Shapiro-Wilk test. Normally distributed data were expressed as mean ± standard deviation. For continuous outcomes, one-way ANOVA was used for comparing a single independent variable across multiple groups, followed by Tukey’s HSD *post hoc* tests (which control the family-wise error rate). For longitudinal outcomes, repeated-measures ANOVA was used; sphericity was assessed using Mauchly’s test, and when the sphericity assumption was violated, the Greenhouse–Geisser correction was applied to the degrees of freedom, followed by Tukey’s *post hoc* tests. For categorical outcomes, the chi-square test or Fisher’s exact test (when expected cell counts were <5) was used. Bonferroni correction was applied to adjust for multiplicity across the secondary outcome measures; the primary outcome (VAS) was tested without adjustment. A statistically significant difference was determined to be present when P < 0.05.

## 3 Results

### 3.1 Participant flow

Between January 22 and 15 October 2025, 128 patients were screened. Eight patients were excluded prior to randomization: four did not meet the inclusion criteria, one declined to take part, and three were ruled out for other causes. The remaining 120 eligible patients were randomly allocated into four groups, with 30 subjects in each arm. No patients dropped out during the study. All randomized participants completed follow-up and were included in the final analysis (Figure 1).

**FIGURE 1:**
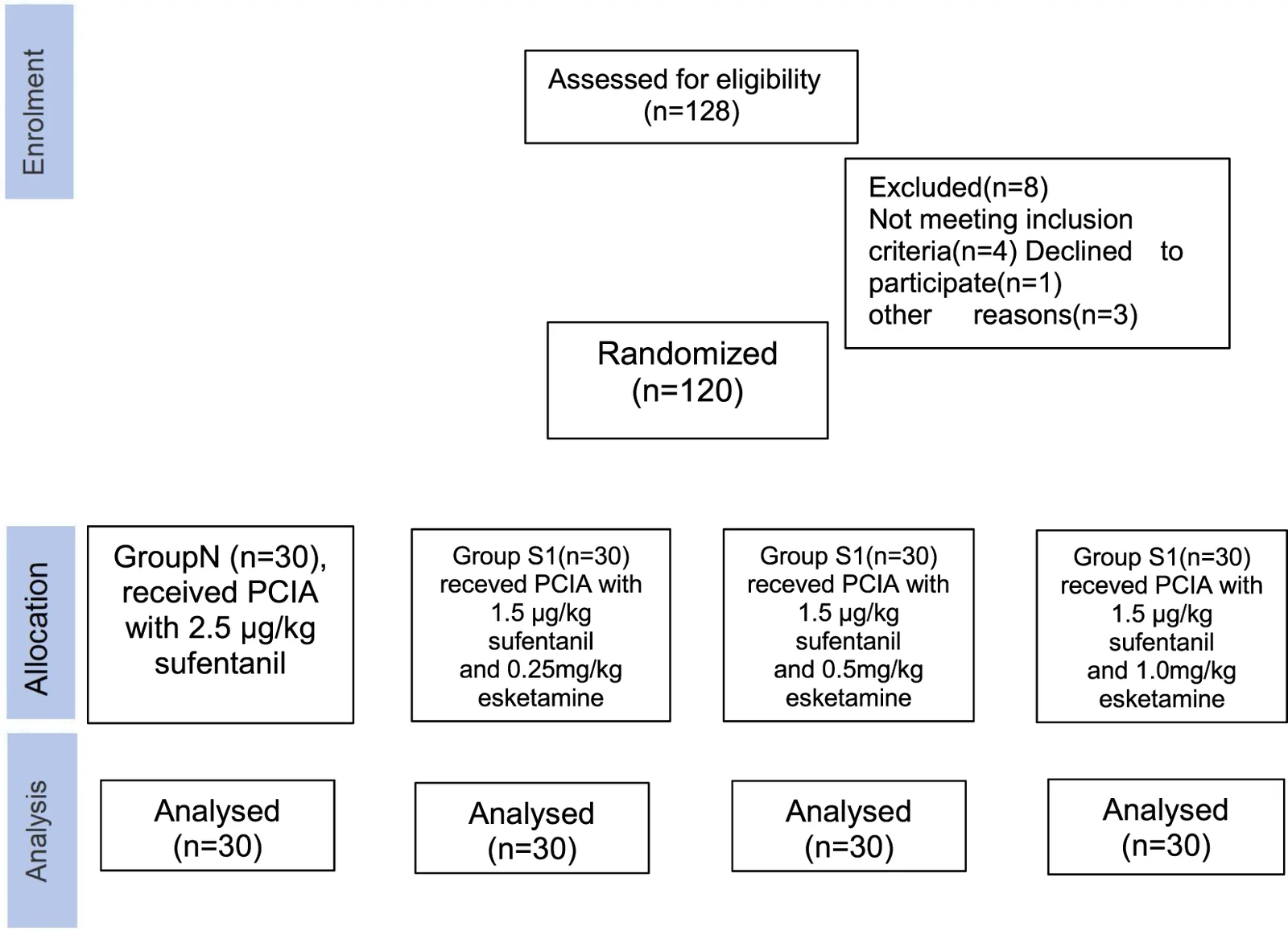
CONSORT flow diagram. From 22 January 2025 to 15 October 2025, a total of 128 patients were screened, of whom 120 were ultimately included in the final analysis.

### 3.2 General information about each group

A statistical comparison of the general characteristics between the four groups was conducted, revealing no statistically significant differences (P > 0.05), as illustrated in Table 1.

**TABLE 1:** Baseline characteristics of the study groups.

Variable		Group N (30)	Group S1 (30)	GroupS2 (30)	GroupS3 (30)	P-value
Gender (Male/Female)		18/12	20/10	19/11	20/10	0.974
Age (years)		48.10 ± 2.84	47.57 ± 3.43	47.47 ± 2.53	47.97 ± 2.62	0.797
BMI (kg/m2)		27.90 ± 3.33	27.57 ± 3.22	27.57 ± 3.66	27.33 ± 3.52	0.937
Surgery duration (min)		94.30 ± 3.50	93.70 ± 3.25	93.50 ± 3.35	93.20 ± 3.48	0.642
Anesthesia duration (min)		124.40 ± 14.19	122.40 ± 15.35	125.27 ± 14.77	128.57 ± 15.54	0.451
Extubation time (min)		10.40 ± 2.76	10.80 ± 3.42	10.90 ± 3.20	10.03 ± 3.61	0.772
ASA classification	I	7 (23.33%)	11 (36.67%)	10 (33.33%)	9 (30.00%)	0.761
	II	23 (76.67%)	19 (63.33%)	20 (66.67%)	21 (70.00%)	

Abbreviations: BMI: Body Mass Index; ASA: american society of anesthesiologists.

### 3.3 Comparison of VAS scores

Two-way repeated-measures ANOVA revealed a significant Time × Group interaction for postoperative VAS scores (F = 8.93, P < 0.001), indicating that the trajectories of pain relief differed among the groups. Simple effect analyses showed that at each postoperative time point (2, 4, 8, and 12 h), the VAS scores in Groups S1, S2, and S3 were significantly lower than those in Group N (all P < 0.05), whereas no statistically significant differences were observed among the three esketamine-containing groups (S1, S2, and S3). These findings are denoted in Table 2; Figure 2.

**TABLE 2:** Comparison of VAS score.

Variable	Time point	VAS score
Group N (30)	2 h postoperatively	2.83 ± 0.38
	4 h postoperatively	3.97 ± 1.16
	8 h postoperatively	4.23 ± 1.52
	12 h postoperatively	3.63 ± 1.33
Group S1 (30)	2 h postoperatively	2.03 ± 0.32^a^
	4 h postoperatively	2.60 ± 0.67^a^
	8 h postoperatively	3.27 ± 1.05^a^
	12 h postoperatively	1.43 ± 0.50^a^
Group S2 (30)	2 h postoperatively	2.00 ± 0.37^a^
	4 h postoperatively	2.50 ± 0.51^a^
	8 h postoperatively	3.13 ± 0.94^a^
	12 h postoperatively	1.10 ± 0.31^a^
Group S3 (30)	2 h postoperatively	1.93 ± 0.37^a^
	4 h postoperatively	2.47 ± 0.57^a^
	8 h postoperatively	3.00 ± 1.02^a^
	12 h postoperatively	1.10 ± 0.31^a^
Time main effect (F, P, ηp^2^)	F = 65.21, P < 0.001, ηp^2^ = 0.574	
Group main effect (F, P, ηp^2^)	F = 52.34, P < 0.001, ηp^2^ = 0.473	
Time × group interaction (F, P, ηp^2^)	F = 8.93, P < 0.001, ηp^2^ = 0.239	

^a^ *P* < 0.05 compared to Group N.

**FIGURE 2:**
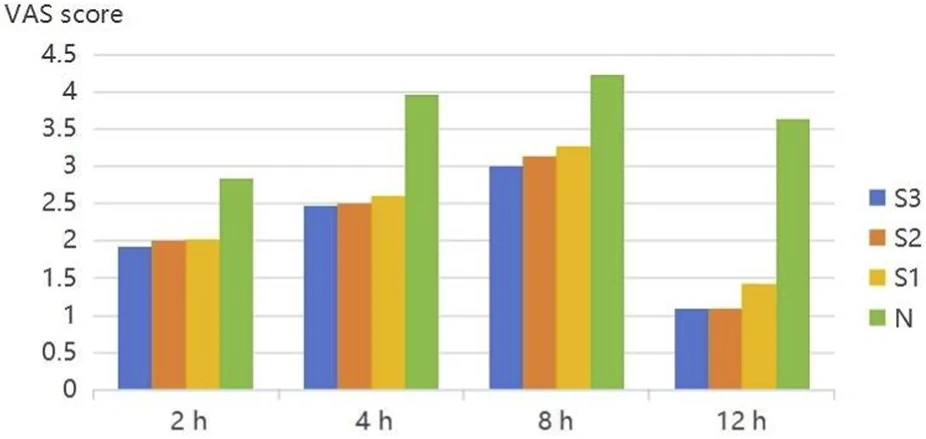
Comparison of VAS scores among the four groups at 2, 4, 8, and 12 h postoperatively.

### 3.4 Comparison of postoperative analgesic consumption and rescue analgesia among groups

The number of rescue analgesia cases was numerically lower in each esketamine-containing group than in the control group, although this difference was not statistically significant. Sufentanil consumption, effective press attempts, and total press attempts in Groups S3, S2, and S1 were significantly lower than those observed in Group N (P < 0.05), as illustrated in Table 3. Compared with the control group, the reduction in 24-h sufentanil consumption in the esketamine-containing groups showed clinically meaningful effect sizes (Cohen’s d of −0.53 in S1, −0.67 in S2, and −0.99 in S3), suggesting a graded opioid-sparing effect with increasing dose; however, these between-dose differences were not formally tested. Importantly, this opioid-sparing effect was achieved without compromising analgesic efficacy, as VAS pain scores were significantly lower in all esketamine-containing groups at all postoperative time points.

**TABLE 3:** Comparison of postoperative analgesic consumption and rescue analgesia among groups.

Variable	Sufentanil dosage (µg)	Effect size	Effective press (times)	Total presses (times)	Cases requiring rescue analgesia
Group N (30)	67.03 ± 4.37	—	3.90 ± 1.14	5.13 ± 1.20	3 (10.00)
Group S1 (30)	65.00 ± 3.06^a^	−0.53	3.20 ± 1.16^a^	4.20 ± 1.27^a^	2 (6.67)
Group S2 (30)	64.40 ± 2.66^a^	−0.67	2.97 ± 0.99^a^	4.17 ± 1.18^a^	1 (3.33)
Group S3 (30)	63.57 ± 2.27^a^ ^,^ ^b^	−0.99	2.50 ± 0.51^a^	3.93 ± 1.11^a^	1 (3.33)
*F/* χ^2^ *-*value	6.415	—	10.498	5.915	1.601
*P-*value	<0.001	—	<0.001	0.001	0.834

^a^ *P* < 0.05 compared to Group N.

^b^ *P* < 0.05 compared to Group S1.

### 3.5 Comparison of Ramsay scores among groups

The two-way repeated-measures ANOVA revealed a significant main effect of Time and Group, as well as a significant Time × Group interaction for Ramsay sedation scores (all P < 0.001; Figure 3). Post-hoc simple effect analyses demonstrated a dose-dependent enhancement of sedation. Specifically: (1) the high-dose group (S3) consistently exhibited the highest sedation scores, significantly surpassing both the medium- (S2) and low-dose (S1) groups at most time points (P < 0.05); (2) the medium-dose group (S2) showed intermediate sedation levels, generally higher than the low-dose group (S1) during the mid-postoperative period; and (3) all three intervention groups (S1, S2, S3) provided greater sedation than the control group (N) at various time points, as detailed by superscript letters in Table 4.

**FIGURE 3:**
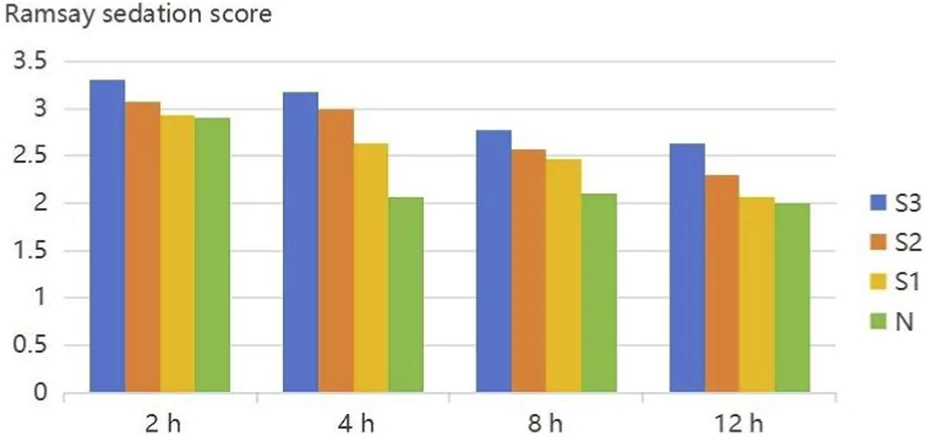
Comparison of Ramsay sedation scores among the four groups at 2, 4, 8, and 12 h postoperatively.

**TABLE 4:** Comparison of Ramsay scores among groups.

Variable	Time point	RSS
Group N (30)	2 h postoperatively	2.90 ± 0.25
	4 h postoperatively	2.07 ± 0.67
	8 h postoperatively	2.10 ± 0.31
	12 h postoperatively	2.00 ± 0.02
Group S1 (30)	2 h postoperatively	2.93 ± 0.31
	4 h postoperatively	2.63 ± 0.67^a^
	8 h postoperatively	2.47 ± 0.51^a^
	12 h postoperatively	2.07 ± 0.48
Group S2 (30)	2 h postoperatively	3.07 ± 0.32
	4 h postoperatively	3.00 ± 0.83^a^ ^,^ ^c^
	8 h postoperatively	2.57 ± 0.46^b^
	12 h postoperatively	2.30 ± 0.37
Group S3 (30)	2 h postoperatively	3.30 ± 0.34^a^ ^,^ ^b^ ^,^ ^c^
	4 h postoperatively	3.17 ± 0.79^a^ ^,^ ^b^ ^,^ ^c^
	8 h postoperatively	2.77 ± 0.47^a^ ^,^ ^b^ ^,^ ^c^
	12 h postoperatively	2.63 ± 0.50^a^ ^,^ ^b^ ^,^ ^c^
Time main effect (F, P, ηp^2^)	F = 35.42, P < 0.001, ηp^2^ = 0.284	
Group main effect (F, P, ηp^2^)	F = 28.15, P < 0.001, ηp^2^ = 0.247	
Time × group interaction (F, P, ηp^2^)	F = 9.86, P < 0.001, ηp^2^ = 0.190	

^a^ *P* < 0.05 compared to Group N.

^b^ *P* < 0.05 compared to Group S1.

^c^ *P* < 0.05 compared to Group S2.

Abbreviations: Ramsay sedation score.

### 3.6 Comparison of oxidative stress indicators among groups

The inter-group comparison revealed no statistically significant differences in preoperative SOD, MDA, GLU, and COR levels across all groups (P > 0.05). At 4 h postoperatively, GLU, COR, and MDA levels were elevated, while SOD levels declined in all groups (P < 0.05). Furthermore, at 4 h postoperatively, Groups S3, S2, and S1 showed higher SOD levels and lower MDA, GLU, and COR levels in comparison to Group N (P < 0.05). However, no statistically significant differences in SOD, MDA, GLU, and COR levels were observed among Groups S3, S2, and S1 at 4 h postoperatively (P > 0.05). These findings are denoted in Table 5.

**TABLE 5:** Comparison of oxidative stress indicators among groups ( $\bar{x}\pm s$ ).

Variable	Indicators	SOD (U/mL)	MDA (nmol/L)	GLU (mmol/L)	COR (mmol/L)
Group N (30)	Preoperative	88.09 ± 6.73	5.24 ± 1.29	4.67 ± 1.04	426.63 ± 79.96
	4 h postoperatively	60.72 ± 9.36	9.31 ± 0.77	5.95 ± 1.47	535.13 ± 95.47
	Change (post-pre)	−27.37 ± 10.98	4.07 ± 1.41	1.28 ± 1.69	108.50 ± 113.22
Group S1 (30)	Preoperatively	88.68 ± 6.68	5.53 ± 1.39	4.58 ± 1.05	426.64 ± 80.84
	4 h postoperatively	68 ± 10.33 ^#^	7.16 ± 0.62 ^#^	5.19 ± 1.07 ^#^	476.89 ± 87.55 ^#^
	Change (post-pre)	−20.68 ± 11.15*	1.63 ± 1.43*	0.61 ± 1.28*	50.25 ± 106.78*
Group S2 (30)	Preoperatively	88.71 ± 6.6	5.53 ± 1.40	4.6 ± 1.06	426.67 ± 80.83
	4 h postoperatively	68.03 ± 10.36^#^	7.15 ± 0.56^#^	5.25 ± 1.04^#^	476.9 ± 87.54^#^
	Change (post-pre)	−20.68 ± 11.25*	1.62 ± 1.39*	0.65 ± 1.26*	50.23 ± 106.70*
Group S3 (30)	Preoperatively	88.79 ± 6.68	5.59 ± 1.47	4.61 ± 1.08	426.73 ± 80.91
	4 h postoperatively	68.09 ± 10.41^#^	7.16 ± 0.62^#^	5.26 ± 1.08^#^	477 ± 87.62^#^
	Change (post-pre)	−20.70 ± 11.30*	1.57 ± 1.48*	0.65 ± 1.32*	50.27 ± 106.88
Statistics	Time main effect (F, P, ηp^2^)	F = 215.34, P < 0.001 ηp^2^ = 0.799	F = 595.41, P < 0.001 ηp^2^ = 0.916	F = 45.21, P < 0.001 ηp^2^ = 0.447	F = 58.33, P < 0.001 ηp^2^ = 0.518
	Group main effect (F, P, ηp^2^)	F = 1.52, P = 0.215 ηp^2^ = 0.040	F = 1.88, P = 0.138 ηp^2^ = 0.050	F = 0.09, P = 0.966 ηp^2^ = 0.003	F = 0.03, P = 0.993 ηp^2^ = 0.01
	Interaction (Time × Group)	F = 5.421, P < 0.001 ηp^2^ = 0.162	F = 85.321, P < 0.001 ηp^2^ = 0.758	F = 3.012, P = 0.033 ηp^2^ = 0.098	F = 3.891, P = 0.011 ηp^2^ = 0.125

#P < 0.05 compared to Group N; * Significant difference compared with the preoperative value within the same group, P < 0.05 (paired t-test from simple effect analysis).

Abbreviations: SOD, superoxide dismutase; MDA, malondialdehyde; GLU, blood glucose; COR, cortisol.

### 3.7 Comparison of adverse events among groups

The incidence rates of agitation, nausea, vomiting, drowsiness, respiratory depression, and overall adverse reactions at 24 h postoperatively did not reveal any statistically significant differences between the groups (P > 0.05). However, Group S3 demonstrated significantly higher rates of psychiatric symptoms (20.0% vs. 6.67% in S1, P = 0.032; vs. 0% in N, P = 0.012) and hypotension (13.33% vs. 3.33% in S1, P = 0.038) when compared with the other groups (P < 0.05). These findings are outlined in Table 6.

**TABLE 6:** Comparison of adverse events at 24 h postoperatively among groups.

Group	Agitation	Nausea and vomiting	Drowsiness	Respiratory depression	Psychiatric symptoms(dissociation or nightmares)	Hypotension	Total incidence
Group N (30)	3 (10.00)	6 (20.00)	5 (16.67)	2 (6.67)	0 (0.00)	0 (0.00)	16 (53.33)
Group S1 (30)	1 (3.33)	3 (10.00)	2 (6.67)	1 (3.33)	2 (6.67)	1 (3.33)	10 (33.33)
Group S2 (30)	2 (6.67)	4 (13.33)	3 (10)	1 (3.33)	1 (3.33)^a^	1 (3.33)	12 (40.00)
Group S3 (30)	1 (3.33)	2 (6.67)	1 (3.33)	1 (3.33)	6 (20.00)^a^ ^,^ ^b^ ^,^ ^c^	4 (13.33)^a^ ^,^ ^b^ ^,^ ^c^	15 (50.00)
*Inter-group P-value*	0.621	0.384	0.275	0.834	0.021	0.043	0.384

^a^ *P* < 0.05 vs. Group N.

^b^ *P* < 0.05 vs. Group S1.

^c^ *P* < 0.05 vs. Group S2.

## 4 Discussion

### 4.1 Background and rationale

Lumbar decompression surgery is a widely performed spinal procedure used to address lumbar spine disorders by alleviating nerve root compression, reducing pain, and enhancing functionality. This surgical intervention is frequently associated with considerable postoperative discomfort, including pain and soreness, necessitating effective anesthesia management for optimal recovery (Suzuki and Nakamura, 2022; Zhang et al., 2021; Li et al., 2021). Clinical evidence indicates that the quality of anesthesia achieved with conventional general anesthesia in lumbar decompression surgery is often insufficient. This insufficiency may be explained by individual variability in response to anesthetic agents, which can arise from differences in factors such as physical constitution, age, and preexisting medical conditions. As a result, standard anesthetic dosing may fail to meet the requirements of all patients, leading to suboptimal recovery outcomes for certain individuals.

### 4.2 Overall findings: a dissociation between analgesic benefit and dose-dependent toxicity

This study found that the addition of low-dose esketamine (0.25–1.0 mg/kg) to a reduced-sufentanil PCIA regimen significantly improved postoperative analgesia and reduced opioid consumption in patients undergoing lumbar decompression surgery (Qiu et al., 2022; Nikayin et al., 2022). Because the esketamine-containing regimens employed a lower sufentanil dose than the control regimen, these effects reflect the combined esketamine-plus-reduced-opioid regimen rather than esketamine in isolation. Our central finding reveals a clinically relevant pharmacological phenomenon: within this dose range, the analgesic and opioid-sparing benefit showed no statistically significant incremental gain above 0.25 mg/kg, whereas neuropsychiatric and cardiovascular adverse reactions (e.g., sedation, dissociative symptoms, hypotension) increased in a dose-dependent manner (Wang MR. et al., 2023; Silverstein et al., 2021). This pattern—broadly consistent with prior reports on the perioperative use of ketamine and esketamine for analgesia, sedation, and their dose-related effects (Mion and Himmelseher, 2024; Sedigh et al., 2026; Sangi et al., 2025c; Sangi et al., 2025a; Aligholizadeh et al., 2026; Sangi et al., 2025b)—raises an important clinical question: why do the therapeutic effects and the adverse-effect profile of the same drug exhibit apparently different dose–response relationships?

### 4.3 Analgesic benefit and opioid-sparing effect

Our data consistently demonstrate the analgesic efficacy of the esketamine-containing regimens. Sufentanil consumption was significantly reduced (by approximately 3.0%–5.2%) in all esketamine-containing groups, and VAS scores were significantly lower at all postoperative time points compared with the control group. Importantly, no statistically significant incremental analgesic benefit was observed across the four-fold dose range from 0.25 to 1.0 mg/kg. We emphasize that the absence of a statistically significant between-dose difference does not establish equivalence; no formal equivalence or non-inferiority analysis with a predefined clinical margin was performed, and a dedicated dose-ranging equivalence trial would be required to confirm a true analgesic plateau. These observations are consistent with the opioid-sparing effects of ketamine reported in the broader perioperative literature, including its use in spinal anesthesia and its influence on sedation and postoperative nausea and vomiting (Sangi et al., 2025c; Sangi et al., 2025). Mechanistically, the analgesic effect is thought to arise from antagonism of the NMDA receptor, which may inhibit the glutamate-mediated facilitation of nociceptive signaling, thereby attenuating central sensitization and hyperalgesia and delaying the development of opioid tolerance (Zipursky et al., 2021; Mion and Himmelseher, 2024); however, these mechanisms were not directly investigated in the present study.

### 4.4 Dose-dependent adverse events and potential mechanisms

In contrast to the apparent plateau in analgesic benefit, the safety profile showed a clear dose-dependency: the highest-dose group (1.0 mg/kg) exhibited a significantly higher incidence of hypotension and dissociative symptoms and higher Ramsay sedation scores (Hou et al., 2025; Evans et al., 2024; Chevalier et al., 2025; Ragone et al., 2024). This divergence could plausibly reflect the heterogeneous distribution and function of NMDA receptors within the central nervous system. At low doses, the drug may preferentially occupy hyperactive NMDA receptors in pain pathways (e.g., the spinal dorsal horn) heightened by surgical trauma, achieving analgesia with relatively little impact on brain regions governing cognition, emotional integration, and cardiovascular regulation. As the dose increases, receptor occupancy may rise non-linearly, leading to more widespread inhibition of areas such as the prefrontal cortex, limbic system, and brainstem cardiovascular centers, which could account for the dose-limiting neuropsychiatric and cardiovascular effects (Lengvenyte et al., 2022). We present this as a possible explanation, not a demonstrated mechanism. The dose-related emergence of dissociative symptoms observed here is consistent with previous reports describing the neuropsychiatric profile of ketamine and esketamine (Evans et al., 2024; Chevalier et al., 2025; Ragone et al., 2024; Sedigh et al., 2026).

### 4.5 Oxidative and surgical stress markers

Beyond analgesia, biochemical markers indicated that oxidative stress (SOD, MDA) and surgical stress responses (COR, GLU) at 4 h postoperatively were significantly attenuated in all esketamine-containing groups compared with the control group. This finding is consistent with a reduction in oxidative and surgical stress associated with the esketamine-containing regimen; however, these marker changes do not by themselves demonstrate organ protection, and the term “multi-organ protection” has therefore been avoided. Possible mechanisms, not directly measured here, could include anti-inflammatory and immunomodulatory effects—for example, inhibition of the NLRP3 inflammasome or modulation of MAPK/NF-κB signaling, as suggested by preclinical work (Cai et al., 2024; Jiang et al., 2024)—and potential neurotrophic signaling via BDNF–TrkB pathways (Jiang et al., 2024). The moderate reduction in cortisol also raises the possibility of a favorable modulation of the hypothalamic–pituitary–adrenal axis rather than complete suppression, but this remains speculative and requires dedicated investigation (Han et al., 2025).

### 4.6 Preliminary efficacy-safety profile

Taken together, this study provides preliminary evidence regarding the efficacy-safety profile of the investigated esketamine doses rather than defining a therapeutic window. Within the tested range, the analgesic and opioid-sparing benefit was similar across doses, whereas sedation, hypotension, and psychomimetic symptoms increased with dose. Consequently, the lowest dose tested (0.25 mg/kg) may offer a favorable efficacy-safety balance, but this interpretation is provisional and should be confirmed in larger, adequately powered dose-ranging trials that include a formal equivalence or non-inferiority analysis and, ideally, a matched-opioid control arm (Hou et al., 2025; Han et al., 2025; Han et al., 2023).

### 4.7 Comparison with previous studies

The present study showed that the esketamine-containing regimens significantly reduced the need for rescue analgesics, although the reduction in total sufentanil consumption was modest. By contrast, a previous trial of esketamine for PCIA after cesarean section reported more prominent reductions in sufentanil consumption at 6 and 24 h postoperatively (Han et al., 2023). This discrepancy may be explained by differences in analgesic pump background infusion settings, the severity of surgical trauma, and inter-population variability in drug metabolism. Consistent with our results, esketamine administration did not elevate the overall incidence of adverse events relative to the control group. The dose-dependent increase in dissociative symptoms and sedation observed here also aligns with the broader literature on the dose-related effects of ketamine (Mion and Himmelseher, 2024; Sedigh et al., 2026; Sangi et al., 2025a).

### 4.8 Limitations

This work has several notable limitations. First and most importantly, the study was not designed to isolate the independent effect of esketamine: the esketamine-containing regimens used a lower sufentanil dose (1.5 μg/kg) than the control regimen (2.5 μg/kg) in order to test the opioid-sparing hypothesis. Consequently, the observed differences in analgesia and adverse outcomes reflect the combined esketamine-plus-reduced-opioid regimen and cannot be attributed to esketamine alone; a matched-opioid control arm or an add-on design with identical opioid doses would be needed to isolate the esketamine-specific effect. Second, no formal equivalence or non-inferiority analysis with a predefined margin was performed, so the absence of a statistically significant between-dose difference in analgesia cannot be interpreted as evidence of a true ceiling effect. Third, we adopted only three esketamine dose gradients (0.25, 0.5, and 1.0 mg/kg) without testing lower concentrations, so the minimum effective dose could not be determined. Fourth, all participants were recruited from a single center with a relatively small sample size, limiting generalizability and power to detect rare or mild adverse events. Fifth, follow-up was limited to 24 h postoperatively without long-term outpatient follow-up, so delayed adverse reactions could not be recorded. Sixth, we relied on intermittent clinical scoring rather than continuous monitoring such as capnography, so minor transient respiratory suppression might be underestimated.

Regarding outcome measurement, we acknowledge that no validated quality-of-recovery instrument (e.g., QoR-15 or QoR-40) was administered; accordingly, the original title phrase “quality of post-anesthetic recovery” has been replaced with precise descriptors of the outcomes actually measured (postoperative analgesia and opioid consumption). Sedation was assessed with the Ramsay Sedation Scale; although agitation was assessed in parallel with the Riker Sedation-Agitation Scale, the Richmond Agitation-Sedation Scale (RASS) would have provided a more contemporary, unified assessment, and its absence is acknowledged. Although the VAS is the standard primary efficacy endpoint in acute postoperative pain trials, it remains a subjective, patient-reported measure; this subjectivity was mitigated by blinded assessment at fixed postoperative time points but is nonetheless acknowledged as a limitation.

Regarding blinding and registration, although patients, anesthesiologists, outcome assessors, and data analysts were blinded, the dose-dependent sedation and dissociative symptoms in the higher-dose groups may have allowed partial unblinding of assessors, which we acknowledge as a limitation. Finally, regarding trial registration, several exploratory indicators were initially listed as co-primary endpoints on the registry system, whereas the sample size was calculated solely on the basis of the VAS pain score because adequate published reference data were lacking for the other markers. The VAS was confirmed as the primary outcome before data analysis, and the registry has been amended to designate the VAS as the sole primary outcome with all other endpoints reclassified as secondary or exploratory, in accordance with the CONSORT reporting guidelines. Multicenter trials with longer follow-up, matched-opioid control arms, formal equivalence/non-inferiority analyses, and continuous vital sign monitoring are needed to confirm our current results.

## Data Availability

The raw data supporting the conclusions of this article will be made available by the authors, without undue reservation.
